# Applications and Potentials of a Silk Fibroin Nanoparticle Delivery System in Animal Husbandry

**DOI:** 10.3390/ani14040655

**Published:** 2024-02-19

**Authors:** Yiyao Guo, Mian Muhammad Awais, Shigang Fei, Junming Xia, Jingchen Sun, Min Feng

**Affiliations:** Guangdong Provincial Key Laboratory of Agro-Animal Genomics and Molecular Breeding, College of Animal Science, South China Agricultural University, Guangzhou 510642, China; guo1473064177@163.com (Y.G.); awaismian31@yahoo.com (M.M.A.); fsg514696098@163.com (S.F.); xiajm2018@163.com (J.X.); cyfz@scau.edu.cn (J.S.)

**Keywords:** silk fibroin, nanoparticles, drug delivery, animal husbandry

## Abstract

**Simple Summary:**

Silk fibroin (SF) from silkworms is exceptionally biocompatible and biodegradable. The nanoparticle delivery systems constructed with SF can improve drug delivery and enhance action efficiency. This review outlines the various nanoparticle delivery systems, focusing on the application and potential of eco-friendly SF as a delivery system in animal husbandry. The aim is to provide theoretical references for the further development and application of SF in animal husbandry.

**Abstract:**

Silk fibroin (SF), a unique natural polymeric fibrous protein extracted from *Bombyx mori* cocoons, accounts for approximately 75% of the total mass of silk. It has great application prospects due to its outstanding biocompatibility, biodegradability, low immunogenicity, and mechanical stability. Additionally, it is non-toxic and environmentally friendly. Nanoparticle delivery systems constructed with SF can improve the bioavailability of the carriers, increase the loading rates, control the release behavior of the deliverables, and enhance their action efficiencies. Animal husbandry is an integral part of agriculture and plays a vital role in the development of the rural economy. However, the pillar industry experiences a lot of difficulties, like drug abuse while treating major animal diseases, and serious environmental pollution, restricting sustainable development. Interestingly, the limited use cases of silk fibroin nanoparticle (SF NP) delivery systems in animal husbandry, such as veterinary vaccines and feed additives, have shown great promise. This paper first reviews the SF NP delivery system with regard to its advantages, disadvantages, and applications. Moreover, we describe the application status and developmental prospects of SF NP delivery systems to provide theoretical references for further development in livestock production and promote the high-quality and healthy development of animal husbandry.

## 1. Introduction

As animal husbandry plays an integral role in national economies, continuous outbreaks of livestock and poultry diseases during breeding pose significant challenges to animal health and food security [1]. The devastating African swine fever (ASF) outbreak in 2018 posed a significant threat to the pig industry, with a high mortality rate, and caused substantial economic losses to the pig industry [2,3]. Frequent outbreaks of ASF, avian influenza (AI), lumpy skin disease (LSD), porcine reproductive and respiratory syndrome (PRRS), foot-and-mouth disease (FMD), African horse sickness (AHS), and others have also caused huge economic losses to animal husbandry worldwide [4,5,6,7,8,9,10]. Effective prevention, control, and treatment of major animal diseases remain a big hurdle, and delivering drugs using nanotechnology for disease prevention is emerging as a promising technique.

In recent years, the application of nanotechnology in drug treatment or drug delivery has gradually emerged. The use of nanoparticles (NPs) for drug delivery to improve the efficiency of drug action and reduce side effects has become a trend in drug therapy [11]. However, some NPs in traditional delivery systems have shown toxic effects, such as inhibiting plant lateral root growth, affecting development, decreasing growth rate, causing tissue damage, and inducing hepatotoxicity and neurotoxicity in the plants, cell lines, and animal models [12,13,14]. Studies using zebrafish as a model have shown that long-term exposure to different concentrations of titanium dioxide NPs had adverse effects on aquatic organisms. It was revealed that titanium dioxide NPs could translocate between zebrafish organs and were toxic to heart tissues by traversing the blood–brain and blood–heart barriers [15]. Given the potential risks of NPs, it is necessary to transform existing NPs or find new ones to solve these problems while benefiting human society.

Silk fibroin (SF) is the main component of the silkworm cocoon, historically valued for its strength and luster. Recently, it has gained attention as a biomaterial due to its ability to interact favorably with biological systems and naturally degrade and its lack of cytotoxic effects. SF consists of three subunits—the heavy chain (H-chain), light chain (L-chain), and P25 glycoprotein chain [16]. As a natural organic polymeric biomaterial made up of 18 amino acids, including glycine (43%), alanine (29%), and serine (12%) [17]. SF’s unique amino acid composition and spatial structure allow it to have flexible molecular chains, excellent biocompatibility, low immunogenicity, low allergenicity, and ecofriendly features, making it an effective candidate for drug delivery [18]. Notably, silk fibroin nanoparticles (SF NPs) have many modifiable sites, which can be used alone or in combination with various drug delivery strategies [19]. Therefore, NP delivery systems constructed with natural materials like SF are emerging as an exciting tool.

In this paper, we particularly focus on the potential future application of the SF NP delivery system in animal husbandry, summarize its application, and compare its advantages and disadvantages with other NP delivery systems, aiming to provide theoretical ideas and a foundation for the advancement of the SF NP delivery system in animal husbandry.

## 2. NP Delivery System

The International Organization for Standardization (ISO) defines “nanomaterials” as “materials with any nanoscale external dimensions or with nanoscale internal structure or surface structure”. NPs, on the other hand, are nano-objects with all three external dimensions at the nanoscale. NPs are tiny particles ranging in size from approximately 1–100 nm in diameter. They possess a large surface area ratio and flexibility and exhibit unique biological characteristics [20]. They can serve as a modification platform to efficiently bind, adsorb, and carry anti-cancer drugs such as DNA, RNA, protein, and imaging agents [21,22,23,24]. Over the past several decades, extensive research has been conducted on the use of NPs in drug delivery systems, disease diagnosis, vaccine development, antibiosis, nutrition, imaging tools, the textile industry, cosmetics, and other biological and non-biological fields [25]. NP delivery systems are generally defined as those that use NPs as a delivery vehicle to deliver drugs, enzymes, peptides, vaccine antigens, and other materials in vivo [26]. These delivery systems can protect DNA, effectively enhance the immunogenicity of DNA, minimize toxicity, achieve the targeted delivery of antigen-presenting cells (APCs), enhance DNA uptake and nuclear entry, and improve overall antigen-specific immune responses [27]. Furthermore, NP delivery systems can minimize drug toxicity, increase the solubility of drug formulations, facilitate the crossing of biological barriers, slow down the in vivo metabolic rate of drugs, and effectively improve drug utilization capacity [28,29]. In antitumor treatment, nanomaterial-based delivery systems relying on targeting and enhanced permeability and retention effects (EPR) of anticancer drugs are more effective, safer, and less harmful [30]. Szewczyk et al. suggested that NPs encapsulating doxorubicin and siRNA could potentially be used to develop new treatment method for canine osteosarcoma [31]. The application of nanomaterials and nanotechnology in animal husbandry is very compelling and has already found applications in animal nutrition, feeding and breeding, and animal welfare, especially with respect to NPs’ significant anti-inflammatory and antitumor effects, which greatly improve animal breeding [32]. Applying nanomaterials and nanotechnology in animal husbandry could prevent abrupt outbreaks that cause significant economic losses. Here, we summarize some commonly used NPs, with special emphasis on SF NPs.

Due to the unique drug delivery function of NPs, various types of NPs have been extensively studied and developed for drug delivery in the past decades. Currently, the most common types of NPs used for drug delivery include lipid-based [4,5,6], polymeric micelles [7,8], inorganic materials [33,34], and protein-based [35]. These delivery systems can reach the cellular/molecular level and detect the transmission level of disease in organisms [36].

### 2.1. Lipid Nanoparticle Delivery System

Lipid nanoparticles (LNPs) are a non-viral drug delivery system, including liposomes, solid lipid NPs, and nanostructured lipid carriers. Liposomes are the first nano system approved for protein and drug delivery [37]. LNPs originate from liposomes that deliver small-molecule drugs and are cationic, spherical vesicles. They are composed of phospholipids and cholesterol [38]. They demonstrate high biocompatibility and can enter the cytoplasm through natural biological processes like membrane fusion, owing to the phospholipid bilayer of the plasma membrane [39]. LNPs have been shown to reduce drug toxicity, enhance drug stability, and improve drug bioavailability [40]. Studies have indicated that LNPs are effective immune stimulators in mRNA-based vaccine delivery and enhance the efficacy of protein subunit vaccines [41,42]. Many LNPs-based siRNA drugs have entered the clinical trial stage [43]. However, LNPs also have some disadvantages, such as their complex composition, relatively lower stability, and targeting efficacy [44,45]. Leakage may occur when loaded with drugs, and increasing the dose can have toxic side effects on the organism [46]. Additionally, emulsifiers that produce toxicity to the body further limit the potential application of cationic NPs [47].

### 2.2. Polymer Micelle Nanoparticle Delivery System

Polymer micelle NPs (PM NPs) are nanocarriers consisting of hydrophilic shells and hydrophobic cores formed by the self-assembly of amphiphilic copolymer molecules in an aqueous medium, with particle sizes ranging from 10 to 100 nm [48,49]. The hydrophilic polymer blocks commonly used include polyethylene glycol, poly (2-oxazoline), and poly (amino acid), while the hydrophobic blocks include polyether, polyester, and poly (amino acid). By modulating the ratio of the amphiphilic blocks, the size, morphology, and hydrophobicity of the formed nanocarriers can be controlled [48]. Due to their simple preparation methods, range of available polymers, and ease of modification, PMs are widely used as nanocarriers in anti-cancer research [50]. PM NPs are also generally stable and versatile and can enhance immune response [51,52]. However, PMs also exhibit drawbacks, such as a low drug payload in aqueous environments [53]. Thus, promisingly ideal PM formulations are expected to improve the therapeutic efficacy of drugs and DNA vaccines, as well as detect and treat domestic animal diseases.

### 2.3. Inorganic Material Nanoparticle Delivery System

Inorganic nanomaterials are the easiest to manufacture and can be classified as metals, semiconductors, and nonmetals such as gold, silver, silicon dioxide, titanium dioxide, rare earth oxides, iron oxide, zinc oxide, and quantum dots [54]. These have received widespread attention in biomedicine due to their unique size and properties, including their optical, electrical, and magnetic characteristics [55]. Inorganic NPs with therapeutic functions have been widely developed and used in clinical applications, innovating the field of biomedicine in three major aspects: nano-diagnostics, nano-therapeutics, and regenerative medicine [56]. In recent years, immunotherapy has become a major treatment for cancer [57]. Iron oxide nanoparticles (IONPs) are excellent candidates for immunotherapy because of their high surface energy and chemical reactivity of the nucleus, as well as their magnetic properties, which can enhance magnetic resonance imaging (MRI) techniques [58,59]. In addition, the magnetism of IONPs has been used for cellular permutation immunotherapy and T-cell enrichment, where magnetization of cytotoxic T cells and natural killer (NK) cells can guide the delivery of these cells to specific tumor sites, thereby improving therapeutic efficacy [60,61]. However, a significant disadvantage of these NPs is that they are difficult to biodegrade and potentially accumulate in the body, which can cause long-term toxicity to humans and the environment. Therefore, when applying these NPs, attention should be paid to their immunogenicity and toxicity mechanisms in vitro/in vivo [62].

### 2.4. Protein Nanoparticle Delivery System

Protein-based NP delivery systems utilize proteins with natural physicochemical properties as delivery vehicles to improve the solubility of drugs in the blood and influence the circulation behavior, ultimately increasing the local concentration of the drug at the tumor site [63]. Keratin, a structural protein of ectodermal cells, has been used by Annalisa to prepare keratin NPs containing doxorubicin (DOX) using ionic gelation and an aggregation of keratin extracted from wool [64]. In vitro simulated release experiments showed that the NPs prepared by the ionic gel method were pH-responsive, with a cumulative drug release of about 60% within 24 h in the simulated tumor microenvironment and about 38% within 24 h in the simulated normal body fluid. Multifunctional nanocarriers, such as SF, albumin, keratin [65], zein [66], and soy protein, are of great importance in drug delivery. Among them, SF is an FDA-approved polymer that can be processed into nanoscale particles in a mild environment [67]. Due to its excellent properties, such as a lower inflammatory response at the degradation site than biocompatible synthetic polymers, such as polylactic acid, SF has been a hot topic of research as a new delivery system in recent years [63]. Table 1 summarizes the preparation, characteristics, and applications of several NPs (Table 1). Furthermore, we elaborate in detail the properties of SF. Finally, we summarize the application of the SF delivery system in animal husbandry and discuss the prospects for its development.

## 3. SF NP Delivery System

Silk protein is primarily composed of SF (70~80%) and sericin (20~30%). Silk consists of two monofilaments bonded in parallel, with sericin on the periphery of the cocoon silk, wrapped around the SF fiber surface to form a monofilament. Sericin helps to maintain the structural integrity of the cocoon by binding two SF fibers together and plays a protective and adhesive role for SF [68]. Although sericin has good water solubility, SF is generally insoluble in water [69]. Structurally, SF is composed of a hydrophobic and a hydrophilic block, with a highly conserved repetitive sequence of glycine, alanine, and serine residues in the hydrophobic part and a sequence of side chains and charged amino acids in the hydrophilic part. It can self-assemble to form a strong, elastic material with high mechanical strength and toughness [70]. In addition to its significant mechanical properties, the biodegradation rate of SF can be adjusted by changing its crystallinity, molecular weight, or cross-linking degree [71,72]. Cai et al. treated the SF membrane with different concentrations of ethanol and found that with the increase in ethanol concentration, the amorphous and metastable conformation of the SF membrane changed into a stable crystal β-fold. The adjustable crystallinity indicates that SF is a degradable material [73]. The higher the crystallinity, the lower the biodegradation rate [74]. Fernández-García et al. implanted in situ gelled SF hydrogels into the brains of mice and found that the inflammation and cell death in the implanted areas were only temporary. The mouse also did not show considerable cognitive or sensorimotor deficits, suggesting that SF is safe to use in mice with good tolerability [75]. The characteristic physicochemical properties of SF, such as high strength, resistance to chemicals and microorganisms, and low elasticity and ductility, mostly derive from its crystal structure [68]. As a functional biomaterial, SF plays an essential role in the biomedical field and has been applied to gene therapy, tissue regeneration, skin repair, drug release, and cell encapsulation [76,77,78,79]. Seib et al. successfully loaded DOX onto SF NPs, and the released loaded drug showed good pH responsiveness. In vitro studies on human breast cancer cell lines showed no cytotoxicity of SF NPs, demonstrating that drug-loaded SF NPs can act as anti-cancer drugs that target lysosomes [80]. Furthermore, SF has several reactive amino and tyrosine residues that can be easily modified to add new functions [81]. Liu et al. used 1,3-propane sultone to modify the surface of native SF. They demonstrated that the reaction occurred mainly on serine amino acid residues and increased the crystallinity and β-sheet content of the modified serine proteins [82].

**Figure 1:**
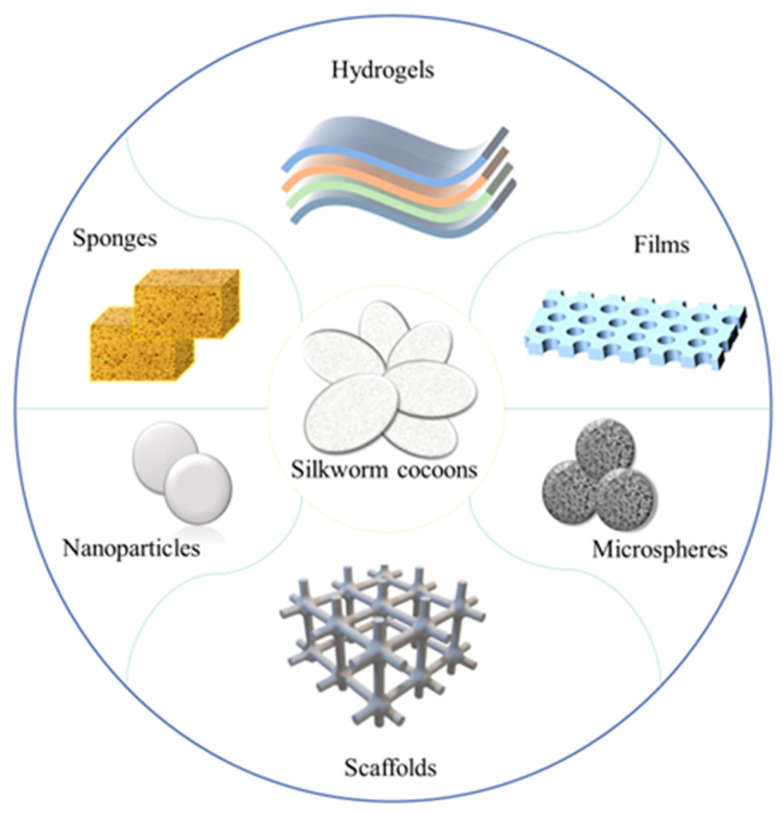
Different forms of SF from silkworm cocoons.

The utilization of SF as a drug delivery material can be achieved by various methods, including the optimization of its primary structure, the incorporation of functional chemical groups, the adjustment of the self-assembly process, and the regulation of the interaction between SF and loading agents [18]. SF can undergo water-soluble and non-water-soluble states under certain conditions, indicating its robust modifiability. Based on different application requirements, SF can be processed into various forms, including hydrogel, film, microsphere, nanoparticles, scaffold, sponge, etc. (Figure 1). In addition, the preparation methods are also varied and selected according to the different forms of SF or specific requirements [83,84]. Its exceptional mechanical properties, good biocompatibility, simple loading process, and flexibility in modification and processing have made SF an excellent nanomaterial candidate for drug delivery using nanomaterials.

## 4. Application and Prospects of SF NP Delivery System in Animal Husbandry

In recent years, SF NP delivery systems have been widely used in biomedical fields, playing an important role in the regenerative repair of different tissues due to their outstanding performance as materials [85]. In addition, SF NP delivery systems are progressively recognized as a promising material, providing new opportunities to overcome the limitations of conventional delivery methods. However, the current research on SF NP delivery systems mainly focuses on biomedical fields, while the research on SF NP delivery systems in animal husbandry is still in its early stages.

### 4.1. Veterinary Vaccine

Vaccination has been a vital intervention to prevent both human and animal infectious diseases since the 1800s [86]. Veterinary vaccines are biological products prepared using microorganisms, their metabolites, structural substances or blood, body fluids, and tissues of infected animals. These biological products induce specific immune protection in animals after inoculation and are safe and effective. Immunization is one of the most effective means of preventing infectious diseases in animals, and safe and effective vaccines are essential tools for improving productivity and combating zoonotic diseases [86]. Based on vaccine targets, veterinary vaccines can be produced against viral, bacterial, protozoal, and multicellular pathogens [87]. Veterinary vaccines, including inactivated viral vaccines and live attenuated and toxoid vaccines, have significantly protected animals from infectious diseases [87]. Efforts are ongoing to develop new vaccines with improved efficacy using various developmental strategies. The use of nanomaterials as carriers for antigen delivery and adjuvants has advanced vaccine development through nanotechnology, thanks to their unique physicochemical properties, such as tunable size and large surface area [88,89,90].

**Figure 2:**
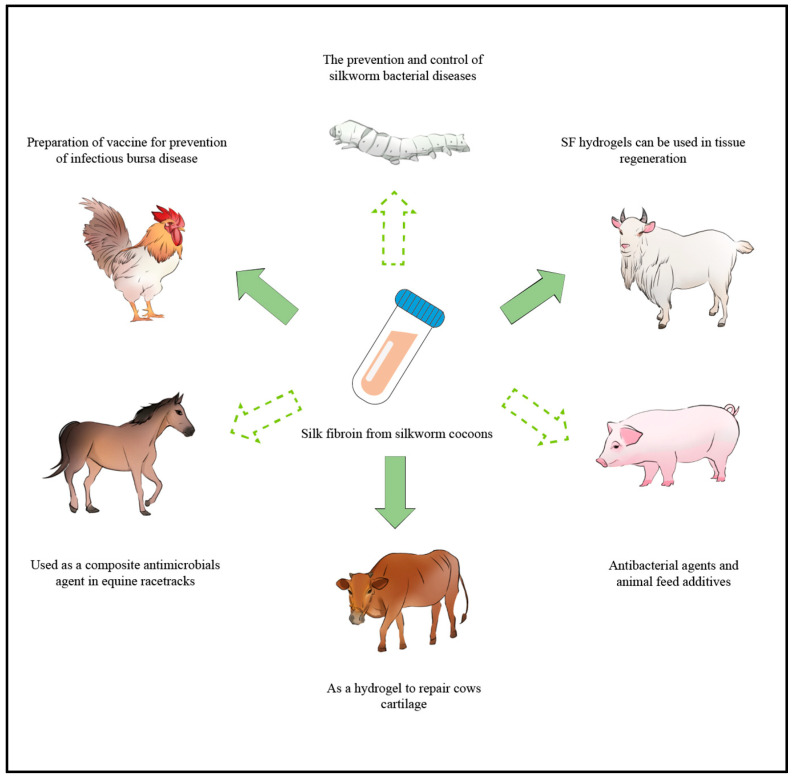
Prospects of the application scenarios of silk fibroin in animal husbandry, such as chickens, silkworms, sheep, pigs, cows, and equines.

The traditional method of preventing infectious bursal disease (IBD) is the vaccination of flocks with inactivated or attenuated vaccines [91]. Attenuated vaccines are more effective than inactivated vaccines, but they pose the risk of residual pathogenicity. Conversely, DNA vaccines express protective antigens after immunization, stimulating the body to produce an immune response, resulting in long-term protection with antigens in their natural form and no risk of infection, making them an option for IBD vaccines. Liu et al. employed microsphere technology in drug delivery and prepared an SF/chitosan DNA composite microsphere vaccine, which stimulated higher anti-infectious bursal disease virus (IBDV) serum ELISA antibodies than chitosan microspheres alone after two weeks of immunization [92]. Moreover, the results of the attack protection experiment in each group showed that the protection rate of the composite microsphere vaccine with added SF was the highest among all groups, reaching 87.5% [92] (Figure 2). Generally, SF is combined with other biological materials to overcome the shortcomings of a single component. Blended SF and chitosan can be crosslinked into a network structure, not only compensating for the drawbacks of chitosan, such as poor toughness and rapid degradation, but is also conducive to the improvement of the brittleness of SF during drying. This results in larger improvements, minimizing morphological differences and mitigating undesirable molding issues and other shortcomings [93].

In recent years, messenger ribonucleic acid (mRNA) vaccines have attracted attention as a new vaccine technology for infectious diseases due to their ease of adapting to new variants, better induction of an immune response, short research cycle, and low production cost [94]. Moreover, mRNA vaccines can induce a specific immune response by expressing antigenic proteins in the cytoplasm, which induces both humoral and cellular immune responses [95]. Furthermore, mRNA vaccines have been considered a relatively safe vaccine format. Researchers have developed different mRNA vaccines against human infectious diseases, such as acute myeloid septicemia, SARS-CoV-2, influenza virus, Zika virus etc. [1,96,97]. To our knowledge, mRNA vaccine research in veterinary vaccines is still in its early stages; there are no relevant reports on the application of veterinary mRNA vaccines. Clinical trials showed that mRNA vaccine-induced antibodies have more targeted binding than those triggered by natural infection [98,99]. Since mRNA is negatively charged and easily degraded by nucleases, commonly used delivery methods include polymer delivery, dendritic cell delivery, and physical delivery by gene [100]. Researchers have studied the stability of the inactivated whole virus RNA of syndrome coronavirus 2 (SARS-CoV-2) in SF matrices, i.e., silk solution and silk film [101]. The findings indicate that it is more stable in silk film than ddH_2_O and silk solution. The SARS-CoV-2 RNA Reference Standard can be stored at room temperature for over 21 weeks, determined by reverse transcription polymerase chain reaction (RT-PCR) assays. Moreover, the silk solution is compatible with RT-PCR, and the relevant substances used in the reaction can be stored at ambient temperature without affecting their activity [101]. In a separate study, researchers encapsulated and stored mRNA in dry SF and found that the mRNA samples could be preserved for over a week at a high temperature of 45 °C [102]. These results offer insights into the preservation of RNA at room temperature and support the use of SF to deliver mRNA. SF may be one of the delivery tools of mRNA vaccines because it protects the RNA, preserves RNA integrity, and plays an important role in its long-term storage and transportation. These mRNA vaccines with SF-based delivery tools have broad application prospects in veterinary vaccines, opening up a new window for research and protecting animals from deadly outbreaks. Also, SF is worth exploring in the development of human mRNA vaccines.

### 4.2. Veterinary Medicine

Traditional veterinary medicines, particularly antimicrobials, are widely employed to control animal diseases. However, antibiotics have become one of the major threats to global public health due to their overuse, which can lead to the development of bacterial resistance. The search for alternatives to antimicrobial products suitable for animal husbandry never stops [103]. Nanotechnology has facilitated the development of non-toxic antibacterial agents for food safety. The use of veterinary drugs based on NP delivery systems on farms has enabled the delivery of drugs directly to target cells, reducing drug doses, residue, and drug use time for economic animals. SF, a medical material certified by the FDA in the United States does not have an apparent antibacterial effect but can be employed as a carrier to deliver natural or synthetic antibacterial materials to achieve antibacterial purposes [104]. Utilizing SF as a carrier to prepare a composite antimicrobial agent can reduce the use of antibiotics and mitigate the emergence of superbugs [105,106].

Fei et al. prepared SF-silver NPs, a composite material with antibacterial and biofilm destruction properties, utilizing the tyrosine residues in SF as the reductant for silver NPs [107]. Despite the easy oxidation characteristics of silver NPs, SF acts as both a dispersant and stabilizer, ensuring the preservation of antibacterial activity. The environment-friendly and cost-effective process of preparing SF-silver NPs relies solely on light as the power source [107]. Furthermore, by utilizing the carrier and anti-inflammatory properties of SF, various forms of SF-based anti-inflammatory agents can be developed by combining different anti-inflammatory agents, such as NPs [108], fibers [109], and hydrogels [110]. Kim et al. found that SF exhibited some anti-inflammatory activity in a mice edema model, and its anti-inflammatory capacity was comparable to that of the pro-freundin derivative PEP-1-FK506BP [111]. Diez-Echave et al. demonstrated the anti-inflammatory properties of SF-quercetin NPs using a dextran sulphate sodium mouse colitis model [29]. Also, SF has been reported as an effective tissue-engineered scaffold to facilitate sheep chondrogenesis or a hydrogel to repair cows’ cartilage [112,113] (Figure 2). The SF-based veterinary medicines not only show the prospect of reducing the risk of antibiotic resistance but also provide a new direction for preparing environmentally safe and highly effective veterinary medication.

### 4.3. Nano-Feed

At the nanoscale, the physical and chemical properties of feed nutrients undergo significant changes due to the increased number of exposed atoms on the surface of the particles. The increased number greatly enhances the nutritional value. It improves the dispersion of nutrients and the shelf life of feed while adsorbing harmful and toxic substances and removing free radicals in animals [114]. This, in turn, reduces the risks of animal diseases, optimizes animal immune status, increases the absorption of nutritional compounds, and improves the quality and safety of animal-derived foods [114]. Moreover, nanotechnology can reduce the demand for preservatives in feed and eliminate the unpleasant odors emitted by feed [115]. Nanomaterials are now recognized as feed additives with potential health benefits. According to relevant studies, adding SF to the diet of gilthead seabream can improve wound healing and increase the expression of important genes involved in tissue repair and extracellular matrix formation. SF is considered an appropriate feed additive [116]. Zinc oxide NPs have been extensively studied as antibacterial agents and animal feed additives in livestock production [117]. Geetha et al. prepared nano zinc oxide by precipitating zinc acetate and investigated its antibacterial and cytotoxic effects as poultry feed additives in vitro. The surface-to-volume ratio of zinc oxide NPs can improve the bioavailability of zinc. The experimental results indicate that zinc oxide NPs have broad-spectrum antibacterial activity, and their production of active oxygen species may be responsible for the antibacterial activity [118]. In addition, the inclusion of copper NPs in feed has been shown to minimize or prevent post-weaning diarrhea in pigs [115] (Figure 2). These reports provide a foundation and direction for future research on the application of SF as a feed additive in animal husbandry. However, although nanotechnology shows great promise in animal feed, medicine, and production, it still requires further testing in industrial practice before it can be fully exploited and utilized.

## 5. Conclusions

While SF NPs have been extensively studied in biomedical fields, their applications in animal husbandry are still in their infancy, and the limited demonstration feasibility hinders their practical applications. Nanotechnology significantly impacts animal production, positively influencing farmed animals in various ways. The use of the SF nano-delivery system in livestock farming will likely provide solutions to problems such as antibacterial-free/antibiotic alternatives and the inefficient delivery of veterinary vaccines, reduce input costs, protect the environment, and improve the output and quality of animal food and farmers’ income.

Moreover, predicting the degradation of SF and the kinetics of drug release based on the SF NP delivery system is challenging due to the variations among different species and individuals within the same species. Introducing controlled-release or pH-responsive materials and adjusting the surface chemistry of the particles allows for more accurate control of the drug release in specific physiological environments, thereby improving the controllability of release. Several issues require solutions, such as systematic biological evaluation, stability, universality and storage, and market regulation [119]. Additionally, reducing the price of silkworm cocoons to achieve low-cost and large-scale production of SF is also a pressing matter to be addressed. Another challenge that must be addressed is the insufficient number of researchers dedicated to the application and research of SF in animal husbandry. Consequently, a considerable journey is ahead before the widespread production and application of the SF NP delivery system can be realized.

The use of SF extracted from silkworm cocoons is both a challenge and a unique opportunity. The SF NP delivery system holds the potential to serve as a carrier for delivering functional ingredients with the capacity to decompose over a specified period. This characteristic offers the prospect of lightening the environmental burden and enhancing the sustainability of animal husbandry. It may contribute to improving the bioavailability of feed or trace elements, thereby aiding in the enhancement of feeding effects, disease prevention, and treatment, ultimately boosting production efficiency through the carriage and controlled release of nutrients. Exploring the application of SF NPs in drug delivery systems presents an avenue for achieving the slow release of hormones and immune enhancers, which, if realized, could potentially reduce the frequency of medication, mitigate environmental drug pollution, enhance drug therapeutic effects, and lower disease incidence. Moreover, there is potential for SF NPs to play a role in wound healing and tissue regeneration, potentially contributing to the reduction of wound inflammation.

In a word, the application of the SF NP delivery system opens up a world full of possibilities. With continuous advancements in biotechnology and nanotechnology, NP delivery systems constructed based on the desirable properties of SF will demonstrate significant potential and find broader applications in animal husbandry. Consequently, the use of SF in animal husbandry will diversify, leading to transformative changes in animal treatment and breeding methodologies.

## Figures and Tables

**Table 1:** Summary of several types of NPs.

Several Types of NPs	Preparation	Characteristics	Applications
LNPs	Numerous methods Simple process Easy to commercialize	Excellent biocompatibility Good drug loading Biodegradable Relatively unstable	Protein and mRNA subunit vaccines Gene therapy
Polymer micelle NPs	A wide range of polymers Simple preparation methods	Excellent biocompatibility	DNA vaccines Hydrophobic anti-cancer drugs
Inorganic NPs	Easy to prepare and store	Good biocompatibility Non-biodegradable	Skin regeneration Antimicrobial therapy Molecular imaging probes
SF NPs	Wide range of sources Simple preparation process Green, safe and non-toxic	Slowly biodegradable Low immunogenicity Easy to chemically modify	Controlled release of drugs Bone tissue regeneration Wound dressings Anti-cancer therapy 3D printing

## Data Availability

No new data were created or analyzed in this study. Data sharing is not applicable to this article.
